# Reasons for and factors associated with issuing sickness certificates for longer periods than necessary: results from a nationwide survey of physicians

**DOI:** 10.1186/1471-2458-13-478

**Published:** 2013-05-16

**Authors:** Richard Bränström, Britt Arrelöv, Catharina Gustavsson, Linnea Kjeldgård, Therese Ljungquist, Gunnar H Nilsson, Kristina Alexanderson

**Affiliations:** 1 Department of Clinical Neuroscience, Division of Insurance Medicine, Karolinska Institutet, Stockholm, Sweden; 2 Department of Public Health Sciences, Karolinska Institutet, Stockholm, Sweden; 3 Stockholm County Council, Stockholm, Sweden; 4 Department of Neurobiology, Care Sciences and Society, Division of Family Medicine, Karolinska Institutet, Stockholm, Sweden

**Keywords:** Sick leave, Sickness certification, Insurance medicine, Physician

## Abstract

**Background:**

Physicians’ work with sickness certifications is an understudied field. Physicians’ experience of sickness certifying for longer periods than necessary has been previous reported. However, the extent and frequency of such sickness certification is largely unknown. The aims of this study were: a) to explore the frequency of sickness certifying for longer periods than necessary among physicians working in different clinical settings; b) to examine main reasons for issuing sickness certificates for longer periods than necessary; and c) to examine factors associated with unnecessary issued sickness certificates.

**Methods:**

In 2008, all physicians living and working in Sweden (a total of 36,898) were sent an invitation to participate in a questionnaire study concerning their sick-listing practices. A total of 22,349 (60.6%) returned the questionnaire. In the current study, physicians reporting handling sickness certification consultations at least weekly were included in the analyses, a total of 12,348.

**Results:**

The proportion of physicians reporting issuing sickness certificates for longer periods than actually necessary varied greatly between different types of clinics, with the highest frequency among those working at: occupational medicine, orthopedic, primary health care, and psychiatry clinics; and lowest among those working in: eye, dermatology, ear/nose/throat, oncology, surgery, and infection clinics. Logistic analyses showed that sickness certifying for longer periods than necessary due to limitations in the health care system was particularly common among physicians working at occupational medicine, orthopedic, and primary health care clinics. Sickness certifying for longer periods than necessary due to patient-related factors was much more common among physicians working at psychiatric clinics. In addition to differences between clinics, frequency of sickness certificates issued for longer periods than necessary varied by age, physicians’ experiences of different situations, and perceived problems.

**Conclusions:**

This study showed that physicians issued sickness certificates for longer periods than actually necessary quite frequently at some types of clinics. Differences between clinics were to a large extent associated with frequency of problems, lack of time, delicate interactions with patients, and need for more competence.

## Background

To get sick-leave benefits, a medical certificate issued by a physician is needed in most countries, in Sweden after seven days of sickness absence [1]. The certificates issued by the physician are important for the decisions made by the employer and the Social Insurance Office regarding the patients’ right to benefits and rehabilitation measures [2]. Handling sickness certification consultations involves a number of tasks for the physician e.g.: to determine if the patient has a disease or an injury; to assess the degree of impairment of function and of the work capacity that is due to that disease or injury; to consider, together with the patient, the pros and cons of being on sick leave; to make decisions regarding duration and grade (full or part time) of sickness absence along with possible investigations, treatments, or other measures – that is, to make a plan of action; to establish adequate contacts with other stakeholders when needed, e.g. concerning contacts with employer regarding demands at work and modification of work tasks; to issues a certificate of adequate quality to provide necessary information for the employer and Social Insurance Office to make decisions regarding entitlement to sickness benefits and return-to-work measures, and to document measures taken [3].

Physicians work with sickness certifications is an understudied field [1]. To date, the majority of studies on physicians’ sickness certification practices have been conducted among primary health care practitioners [1,4]. Several studies and literature reviews have shown a perceived need for more knowledge regarding sickness certification/insurance medicine among physicians [5-10] and a number of perceived problems have been reported e.g. difficulties to assess work capacity and estimate optimal length of sick leave [11]. Recent studies have also identified work with sickness certification as a psychosocial work environmental problem [12]. Further, physicians’ experience of sickness certifying for longer periods than necessary and a perceived medicalization of patients’ non-medical problems has been previously reported [13]. However, the frequency of sickness certifying for longer periods than experienced as necessary in different clinical setting has, to our knowledge, not been reported previously.

The aims of this study were: a) to explore the frequency of sickness certifying for longer periods than actually would be necessary among physicians working in difference clinical settings; b) to examine main reasons for sickness certifying for longer periods than necessary; and c) to examine factors associated with unnecessary issued sickness certificates.

## Method

In the fall of 2008, all physicians living and working in Sweden (a total of 36,898) were sent an invitation to participate in a questionnaire study concerning their sick-listing practices. The questionnaire was to be returned in a prepaid envelope and three reminders were posted to non-respondents. The questionnaire was anonymous and no compensation for participation was offered. A total of 22,349 (60.6%) returned the questionnaire [14]. In the current study, physicians reporting handling sickness certification consultations at least weekly were included in the analyses, a total of 12,348. Information regarding currently active physicians as well as their age, sex, and being a board-certified specialist were obtained from a register covering all physicians in Sweden. The study was approved by the Regional Ethical Review Board of Stockholm (No. 2008/795-31).

### Measures

In addition to archival information regarding participants’ age and gender, answers to the following questionnaire items were included in the analyses.

#### Type of clinic

The participants were asked to indicate at what type of clinic they were mainly working.

#### Frequency of sickness certification

The participants were asked to give an estimate of the frequency by which they had consultations including aspects of sickness certification (more than 20 times per week/6-20 times per week/1-5 times per week/sometimes each month/a few times a year/never or almost never).

#### Frequency of issuing sickness certificates for longer periods than actually would be necessary

Twelve questions were asked to assess the frequency by which the respondents issued sickness absence for longer times than necessary due to different circumstances. The response to each question was indicated on a scale with five alternatives (daily/once a week/once a month/once a year/never or almost never). The responses relating to each of the four main types of situations were combined to give four total measures of issuing unnecessarily long sickness absences due to the following reasons: limitations in the health care system (lack of time for revisits, waiting times for further medical check-ups, or waiting times for other actions/treatments/investigations), wait for action from other stakeholders (waiting time for action from employer, Social Insurance Office, or unemployment office), patient factors (patient does not follow recommendations regarding treatment and rehabilitation), and physician factors (trying to avoid a conflict with the patient, lack of time to discuss alternatives to sick leave during the medical consultations).

#### Organizational support

Two questions were asked regarding organizational support in the sickness certification process. The items concerned: support from the immediate management at the clinic; and whether the clinic had a policy regarding sickness certification aspects.

#### Clinical experiences of sickness certification

Questions regarding specific situations relating to clinical work with sickness certification were asked and the respondents were to indicate the frequency by which they had experienced these situations (more than 10 times per week [4]/6-10 times per week, [3]/1-5 times per week, [2]/sometimes each month, [1]/a few times a year/never or almost never [0]). The questions concerned conflicts with patients, lack of time, and collaborative efforts and referrals to other health care workers or external organizations. The responses relating to each of the three main types of situations were summed and divided by number of items to give three total measures, i.e.: delicate interactions with patients, time restraints, and collaborative efforts and referrals (all sum scores: min = 0; max = 4). In the current study, the Cronbach’s alpha coefficients for the three summed score were 0.79, 0.87, and 0.65, respectively.

#### Perceived problems

A number of specific questions concerned to what extent specific sickness certification tasks were experienced as problematic, with four response alternatives (very [3]/rather [2]/slightly [1]/not at all [0]). The items included the following tasks: assessment of patients’ functional capacity/work capacity; handling differing opinions and communication with the patient; practical issues regarding administration of sickness certificates; and difficulties to follow the new national sick-leave guidelines. The responses relating to each of the four main types of tasks were summed and divided by number of items to give four total measures i.e. problems with assessments, communication, administrative tasks, and to follow the guidelines (all summed scores: min = 0; max = 3). In the current study, the internal consistencies for the four summed score were 0.90, 0.86, 0.80, and 0.85, respectively.

#### Need for more competence

Nineteen items describing potential areas of need for competence in insurance medicine regarding practical handling, communication, responsibilities, and skills regarding sickness certification. The respondents were to indicate the degree to which they felt the need to increase their competence in each of these fields (very high [3]/rather high [2]/small [1]/no [0]). The responses from all items were summed and divided by number of items to give a total measure of perceived need for education (min = 0; max = 3). In the current study, the internal consistency was 0.95. Participants were categorized into two groups based on the summed score and the upper tertile was categorized as “Perceive a need for further competence regarding sickness certification”.

### Statistical analysis

In addition to descriptive statistics of the participants’ responses, a number of logistic regressions were conducted using frequency of issuing sickness absence for longer times than medically necessary as outcome variables. Initial univariate logistic regression analyses were used to assess the association between background variables, work place characteristics, experiences of the sickness certification process, problems relating to sickness certification, and type of clinic, followed by multivariate logistic regression including those variables that were associated with the outcome variable at the p = 0.01 level. Due to the large sample size and high number of statistical tests an alpha-level was set to 0.01. All continuous variables were transformed into Z-scores before being entered into the analyses. All analyses were performed using SPSS version 19.

## Results

### Study sample characteristics

A total of 12,348 physicians were included in the study and distribution between different types of clinics is presented in Table 1. A slight majority of respondents were men (54.1%) and 69.3 percent (8446) were board certified specialists. About one fifth of the respondents (20.9%) responded that there was a well-established policy for handling sickness certifications at their workplace, and 61.0% reported receiving support from their immediate management in their work with sickness certifications.

**Table 1:** Number of participants by clinic and frequency of consultation involving consideration of sickness certification

		**Have sickness certification consultations**	
		**1-5 times a week**	**More than 6 times a week**
	**N (%)**	**N (%)**	**N (%)**
Child and youth	116 (1.0)	90 (77.6)	26 (22.4)
Dermatology	53 (0.5)	46 (86.8)	7 (13.2)
Ear, nose, and throat	359 (3.1)	264 (73.5)	95 (26.5)
Eye	89 (0.8)	76 (85.4)	13 (14.6)
Gynecology	746 (6.1)	462 (61.9)	284 (38.1)
Infection	265 (2.3)	198 (74.7)	67 (25.3)
Internal medicine	1387 (12.0)	972 (70.1)	415 (29.9)
Neurology	226 (2.0)	119 (52.7)	107 (47.3)
Occupational health	463 (4.0)	101 (21.8)	362 (78.2)
Oncology	316 (2.7)	82 (25.9)	234 (74.1)
Orthopedic	865 (7.5)	180 (20.8)	685 (79.2)
Pain	78 (0.7)	20 (25.6)	58 (74.4)
Primary health care	4053 (35.1)	2234 (55.1)	1819 (44.9)
Psychiatry	1024 (8.9)	363 (35.4)	661 (64.6)
Rehabilitation	155 (1.3)	48 (31.0)	107 (69.0)
Rheumatology	181 (1.6)	88 (48.6)	93 (51.4)
Surgery	1176 (10.2)	665 (56.5)	511 (43.5)
**ALL**	11583 (100)	6008 (52.0)	5544 (48.0)

### Frequency of issuing sickness certificates for longer periods than necessary

**Figure 1:**
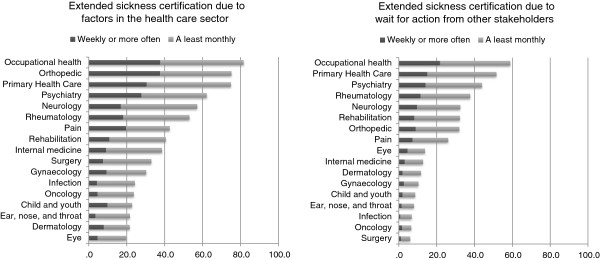
Proportion of physicians working at different clinics regularly issuing sickness absence for longer times than necessary due to limitation in the health care system, or due to waiting time for action from other stakeholders (e.g. employers, Social Insurance Agency).

**Figure 2:**
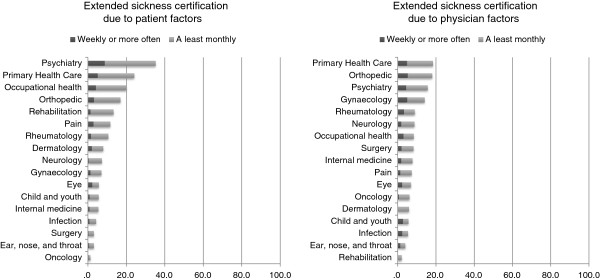
Proportion of physicians working at different clinics regularly issuing sickness absence for longer times than necessary due to patient factors (patient do not follow recommendations regarding treatment and rehabilitation), or physician factors (trying to avoid a conflict with the patient, lack of time to discuss alternatives to sick leave during the medical consultations).

The proportion of physicians reporting sickness certifying for longer periods than necessary varied greatly between physicians working at different types of clinics, see Figures 1 and 2. With the highest frequency among those working at: occupational medicine, orthopedic, primary health care, and psychiatry clinics; and lowest among those working in: eye, dermatology, ear/nose/throat, oncology, surgery, and infection clinics. The most frequent reasons for issuing sickness certificates for longer periods than actually necessary were due to limitations in the health care system such as waiting time for investigation and treatments or lack of treatment options. The least common reasons for issuing sickness certificates for longer periods than necessary were due to physician factors such as to avoid conflicts with patients, or lack of time to discuss alternatives to sick leave with patients.

### Factors associated with extended sickness certificates

The physicians responded to a number of questions regarding experiences related to sickness certification and perceived problems. The responses are presented by type of clinic in Table 2. These variables were then entered into the logistic regression analyses presented in Tables 3 and 4.

**Table 2:** Frequency of reported experiences related to sickness certification, and perceived problems by clinic

	**Frequency of the following situations related to sickness certification**				**Perceived problems regarding sickness certification**			
	**Delicate interactions with patients**	**Lack of time**	**Collaboration, referrals, and support**	**Need for more competence**	**Problems following guidelines**	**General problems**	**Problems with assessment**	**Problems with patient contacts**
	**(min = 0; max = 4)**	**(min = 0; max = 4)**	**(min = 0; max = 4)**	**(min = 0; max = 3)**	**(min = 0; max = 3)**	**(min = 0; max = 3)**	**(min = 0; max = 3)**	**(min = 0; max = 3)**
	**M (SD)**	**M (SD)**	**M (SD)**	**M (SD)**	**M (SD)**	**M (SD)**	**M (SD)**	**M (SD)**
Child and youth	0.40 (0.41)	2.53 (1.10)	0.33 (0.33)	1.22 (0.60)	2.03 (1.27)	1.06 (0.68)	1.13 (0.66)	0.90 (0.66)
Dermatology	0.50 (0.43)	2.31 (1.33)	0.49 (0.37)	1.35 (0.59)	1.62 (1.29)	1.50 (0.83)	1.42 (0.75)	1.25 (0.74)
Ear, nose, and throat	0.43 (0.34)	2.30 (1.29)	0.33 (0.33)	1.22 (0.60)	2.14 (1.20)	1.13 (0.73)	1.29 (0.74)	1.11 (0.73)
Eye	0.50 (0.47)	2.45 (1.23)	0.20 (0.22)	1.11 (0.58)	1.94 (1.28)	1.26 (0.87)	1.26 (0.71)	1.17 (0.79)
Gynecology	0.65 (0.49)	2.40 (1.20)	0.34 (0.29)	1.24 (0.59)	1.69 (1.29)	1.16 (0.71)	1.32 (0.69)	1.24 (0.72)
Infection	0.46 (0.38)	2.32 (1.14)	0.28 (0.26)	1.27 (0.51)	2.07 (1.18)	1.27 (0.63)	1.46 (0.69)	1.21 (0.68)
Internal medicine	0.49 (0.36)	2.57 (1.13)	0.38 (0.33)	1.36 (0.57)	1.75 (1.29)	1.26 (0.71)	1.52 (0.70)	1.27 (0.69)
Neurology	0.55 (0.39)	2.97 (1.03)	0.66 (0.44)	1.35 (0.59)	1.99 (1.25)	1.35 (0.72)	1.52 (0.70)	1.23 (0.69)
Occupational health	0.67 (0.44)	2.11 (1.32)	1.67 (0.50)	1.24 (0.54)	0.83 (1.08)	0.96 (0.77)	1.22 (0.68)	0.90 (0.64)
Oncology	0.36 (0.33)	2.55 (1.17)	0.34 (0.31)	1.16 (0.58)	2.03 (1.27)	1.06 (0.68)	1.13 (0.66)	0.90 (0.66)
Orthopedic	0.76 (0.46)	3.04 (1.06)	0.52 (0.34)	1.24 (0.56)	1.79 (1.28)	1.43 (0.77)	1.48 (0.69)	1.41 (0.72)
Pain	0.64 (0.51)	1.83 (1.49)	1.04 (0.72)	1.15 (0.65)	1.66 (1.27)	1.19 (0.97)	1.35 (0.91)	1.02 (0.83)
Primary health care	0.80 (0.50)	2.96 (0.96)	1.11 (0.46)	1.53 (0.54)	1.33 (1.17)	1.61 (0.73)	1.94 (0.69)	1.57 (0.69)
Psychiatry	0.73 (0.52)	2.73 (1.11)	1.11 (0.61)	1.51 (0.56)	1.73 (1.25)	1.32 (0.79)	1.60 (0.75)	1.23 (0.70)
Rehabilitation	0.46 (0.39)	2.36 (1.22)	1.56 (0.71)	1.35 (0.55)	1.39 (1.25)	1.05 (0.78)	1.16 (0.66)	0.95 (0.66)
Rheumatology	0.59 (0.38)	3.01 (0.96)	0.69 (0.41)	1.36 (0.59)	1.75 (1.24)	1.36 (0.75)	1.60 (0.62)	1.36 (0.68)
Surgery	0.50 (0.40)	2.48 (1.23)	0.25 (0.30)	1.10 (0.59)	1.87 (1.30)	1.09 (0.76)	1.22 (0.73)	1.03 (0.74)
**ALL**	0.65 (0.48)	2.68 (1.16)	0.75 (0.59)	1.36 (0.60)	1.60 (1.27)	1.33 (0.79)	1.55 (0.78)	1.28 (0.75)

**Table 3:** Variables associated with sickness certifying for longer periods than actually necessary due to factors in the health care system or wait for action from other stakeholders

		**Sickness absences issued for longer periods than necessary due to health care factors**		**Sickness absences issued for longer periods than necessary due to wait for action from other stakeholders**	
		**Unadjusted OR**^**a **^**(99% CI)**	**Multivariate**^**b **^**OR (99% CI)**	**Unadjusted OR**^**a **^**(99% CI)**	**Multivariate**^**b **^**OR (99% CI)**
**Gender**	Female	1	-	1	-
	Male	1.06 (0.96-1.16)	-	1.07 (0.96-1.18)	-
**Age**	– 34	1	-	1	1
	35 – 44	0.92 (0.79-1.06)	-	1.47 (1.24-1.75)**	1.26 (0.97-1.63)
	45 – 54	1.00 (0.86-1.16)	-	2.09 (1.76-2.47)**	1.48 (1.09-2.00)*
	55 – 64	1.13 (0.98-1.30)	-	2.51 (2.14-2.95)**	1.78 (1.30-2.44)**
	65 –	1.00 (0.78-1.28)		2.23 (1.70-2.91)**	2.18 (1.39-3.40)**
**Board certified specialist**	No	1	-	1	1
Yes	1.00 (0.90-1.11)	-	1.71 (1.52-1.92)**	1.65 (1.30-2.09)**	
**Sickness certification consultations**					
1-5 times a week		1	1	1	1
More than 5 times a week		2.90 (2.62-3.20)**	1.96 (1.69-2.27)**	2.56 (2.30-2.84)**	1.61 (1.37-1.88)**
**Well established workplace policy**					
Yes (Reference = no)		1.18 (1.05-1.33)**	0.85 (0.71-1.02)	1.56 (1.38-1.76)**	0.94 (0.79-1.13)
**Support from your management**					
Yes (Reference = no)		1.07 (0.97-1.18)	-	0.99 (0.89-1.10)	-
**Frequency of the following situations related to sickness certification:**					
Delicate interactions with patients		3.18 (2.94-3.45)**	1.79 (1.62-1.98)**	2.18 (2.05-2.32)**	1.57 (1.44-1.71)**
Lack of time		2.10 (1.99-2.22)**	1.52 (1.41-1.65)**	1.86 (1.75-1.98)**	1.39 (1.27-1.52)**
Collaboration, referrals, and support		2.47 (2.32-2.64)**	1.62 (1.46-1.80)**	2.80 (2.63-2.99)**	1.78 (1.61-1.97)**
Need for more competence		1.59 (1.51-1.68)**	1.01 (0.92.-1.10)	1.41 (1.34-1.50)**	1.02 (0.93-1.12)
**Perceived problems regarding sickness certification:**					
Problems with the sickness certification guidelines		0.88 (0.83-0.92)**	1.07 (1.00-1.14)	0.86 (0.82-0.90)**	1.05 (0.98-1.12)
General problems with sickness certification		1.87 (1.77-1.97)**	1.17 (1.07-1.29)**	1.62 (1.53-1.71)**	1.04 (0.94-1.14)
Problems with assessments		1.99 (1.88-2.10)**	1.19 (1.06-1.34)**	1.73 (1.63-1.83)**	1.20 (1.05-1.36)**
Problems with patient contacts		1.88 (1.78-1.98)**	1.11 (0.99-1.25)	1.57 (1.48-1.66)**	1.10 (0.98-1.24)
**Clinic**					
Internal medicine		1	1	1	1
Occupational health		7.08 (5.02-10.00)**	4.77 (2.67-8.50)**	9.82 (7.11-13.57)**	2.58 (1.56-4.27)**
Orthopedic		4.82 (3.75-6.19)**	2.53 (1.86-3.46)**	3.23 (2.43-4.29)**	1.52 (1.08-2.15)*
Primary health care		4.74 (3.99-5.63)**	1.85 (1.45-2.35)**	7.32 (5.84-9.17)**	2.43 (1.81-3.26)**
Psychiatry		2.62 (2.10-3.27)**	1.04 (0.77-1.41)	5.38 (4.12-7.03)**	1.93 (1.37-2.73)**
Neurology		2.12 (2.10-3.27)**	1.25 (0.78-2.00)	3.30 (2.15-5.07)**	2.00 (1.20-3.36)*
Rheumatology		1.80 (1.19-2.72)**	0.97 (0.58-1.61)	4.16 (2.65-6.55)**	1.90 (1.09-3.32)*
Pain		1.18 (0.62-2.26)	0.81 (0.32-2.04)	2.43 (1.16-5.07)*	0.70 (0.24-2.11)
Rehabilitation		1.09 (0.69-1.71)	0.39 (0.21-0.72)**	3.27 (1.99-5.38)**	0.86 (0.44-1.66)
Surgery		0.78 (0.63-0.97)*	0.91 (0.69-1.19)	0.44 (0.30-0.64)**	0.52 (0.34-0.81)**
Gynecology		0.69 (0.53-0.89)**	0.50 (0.36-0.70)**	0.80 (0.55-1.16)	0.61 (0.38-0.96)*
Infection		0.51 (0.34-0.76)**	0.55 (0.34-0.90)*	0.51 (0.26-0.98)*	0.48 (0.21-1.10)
Oncology		0.50 (0.34-0.72)**	0.53 (0.34-0.84)**	0.48 (0.26-0.91)*	0.54 (0.26-1.10)
Child and youth		0.47 (0.25-0.88)*	0.60 (0.27-1.35)	0.65 (0.26-1.64)	0.75 (0.26-2.14)
Ear, nose and throat		0.44 (0.31-0.63)*	0.47 (0.30-0.74)**	0.60 (0.34-1.03)	0.52 (0.26-1.02)
Dermatology		0.44 (0.18-1.06)	0.35 (0.11-1.14)	0.92 (0.29-2.86)	1.21 (0.34-4.32)
Eye		0.39 (0.19-0.80)**	0.45 (0.18-1.16)	1.10 (0.48-2.52)	1.75 (0.67-4.56)

^a^ OR denotes odds ratio; CI denotes confidence interval. ^b^ The multivariable analysis was performed by simultaneously entering into the model all variables that were significantly associated with the dependent variable. * = Significant at p < 0.01; ** = Significant at p < 0.001.

**Table 4:** Variables associated with sickness certifying for longer periods than actually necessary due to patient or physician factors

		**Sickness certificates issued for longer periods than necessary due to patient factors**		**Sickness certificates issued for longer periods than necessary due to physician factors**	
		**Unadjusted OR**^**a **^**(99% CI)**	**Multivariate**^**b **^**OR (99% CI)**	**Unadjusted OR**^**a **^**(99% CI)**	**Multivariate**^**b **^**OR (99% CI)**
**Gender**	Female	1	1	1	-
	Male	0.82 (0.72-0.94)**	0.96 (0.80-1.16)	1.12 (0.97-1.29)	-
**Age**	– 34	1	1	1	1
	35 – 44	0.84 (0.69-1.01)	0.83 (0.65-1.07)	0.75 (0.61-0.91)**	0.84 (0.62-1.12)
	45 – 54	0.78 (0.64-0.95)**	0.67 (0.51-0.87)**	0.55 (0.44-0.67)**	0.68 (0.46-1.00)
	55 – 64	0.64 (0.53-0.78)**	0.55 (0.42-0.72)**	0.52 (0.42-0.63)**	0.83 (0.56-1.23)
	65 –	0.44 (0.30-0.66)**	0.55 (0.32-0.93)*	0.29 (0.18-0.47)**	0.54 (0.26-1.10)
**Board certified specialist**		1	**-**	1	1
Yes (Reference = no)		0.69 (0.60-0.79)	**-**	0.57 (0.49-0.65)**	1.04 (0.77-1.41)
**Sickness certification consultations**					
1-5 times a week		1	1	1	1
More than 6 times a week		3,29 (2.85-3.80)**	1.91 (1.57-2.33)**	2.35 (2.02-2.73)**	1.57 (1.27-1.94)**
**Well established workplace policy**					
Yes (Reference = no)		0.94 (0.80-1.10)	**-**	0.58 (0.47-0.70)**	0.56 (0.43-0.75)**
**Support from your management**					
Yes (Reference = no)		1.10 (0.96-1.28)	-	0.95 (0.82-1.10)	-
**Frequency of the following situations related to sickness certification:**					
Delicate interactions with patients		2.52 (2.34-2.70)**	1.74 (1.59-1.90)**	2.88 (2.67-3.11)**	2.12 (1.92-2.34)**
Lack of time		2.04 (1.87-2.23)**	1.42 (1.25-1.62)**	2.22 (2.01-2.45)**	1.34 (1.17-1.54)**
Collaboration, referrals, and support		2.10 (1.96-2.24)**	1.46 (1.30-1.64)**	1.39 (1.30-1.49)**	1.04 (0.91-1.19)
Need for more competence		1.68 (1.56-1.81)**	0.98 (0.88-1.10)	1.99 (1.83-2.16)**	1.17 (1.04-1.32)**
**Perceived problems regarding sickness certification:**					
Problems with the sickness certification guidelines		0.91 (0.85-0.97)**	1.06 (0.96-1.16)	1.07 (0.99-1.15)	-
General problems with sickness certification		1.73 (1.61-1.85)**	0.99 (0.88-1.12)	1.98 (1.84-2.15)**	0.93 (0.82-1.06)
Problems with assessments		2.10 (1.94-2.27)**	1.28 (1.09-1.49)**	2.40 (2.20-2.62)**	1.09 (0.92-1.29)
Problems with patient contacts		2.00 (1.86-2.15)**	1.30 (1.12-1.50)**	2.98 (2.72-3.26)**	2.03 (1.73-2.38)**
**Clinic**					
Internal medicine		1	1	1	1
Occupational health		4.36 (2.84-6.72)**	2.63 (1.34-5.16)**	1.09 (0.65-1.81)	1.28 (0.57-2.89)
Orthopedic		3.56 (2.41-5.24)**	1.99 (1.22-3.24)**	2.62 (1.85-3.71)**	1.24 (0.80-1.95)
Primary health care		5.52 (4.00-7.63)**	2.51 (1.64-3.84)**	2.70 (2.04-3.57)**	1.30 (0.88-1.91)
Psychiatry		9.50 (6.68-13.51)**	6.10 (3.85-9.67)**	2.22 (1.58-3.13)**	1.37 (0.86-2.18)
Neurology		1.36 (0.65-2.84)	1.16 (0.49-2.73)	1.15 (0.59-2-24)	0.92 (0.40-2.14)
Rheumatology		2.07 (1.03-4.16)*	1.83 (0.83-4.06)	1.17 (0.57-2.42)	0.92 (0.39-2.17)
Pain		2.29 (0.83-6.33)	1.35 (0.30-6.20)	0.93 (0.27-3.17)	1.67 (0.37-7.51)
Rehabilitation		2.69 (1.35-5.36)**	2.30 (0.99-5.38)	0.24 (0.05-1.12)	0.39 (0.08-1.96)
Surgery		0.56 (0.33-0.96)*	0.72 (0.39-1.34)	1.08 (0.74-1.58)	1.19 (0.75-1.91)
Gynecology		1.30 (0.80-2.12)	1.22 (0.66-2.23)	1.94 (1.33-2.83)**	1.57 (0.96-2.58)
Infection		0.77 (0.33-1.80)	0.60 (0.18-2.00)	0.68 (0.32-1.44)	0.66 (0.26-1.69)
Oncology		0.23 (0.06-0.86)*	0.38 (0.09-1.52)	0.78 (0.40-1.52)	1.50 (0.68-3.29)
Child and youth		1.04 (0.34-3.20)	1.80 (0.46-7.03)	0.71 (0.23-2.16)	0.93 (0.18-4.88)
Ear, nose and throat		0.56 (0.24-1.31)	0.76 (0.28-2.03)	0.49 (0.23-1.04)	0.56 (0.22-1.37)
Dermatology		1.52 (0.38-6.02)	1.05 (0.13-8.67)	0.75 (0.16-3.53)	0.52 (0.05-5.17)
Eye		1.05 (0.31-3.58)	1.82 (0.39-8.51)	0.87 (0.29-2.68)	0.75 (0.14-3.91)

^a^ OR denotes odds ratio; CI denotes confidence interval. ^b^ The multivariable analysis was performed by simultaneously entering into the model all variables that were significantly associated with the dependent variable. * = Significant at p < 0.01; ** = Significant at p < 0.001.

Issuing sickness certificates for longer periods than necessary due to waiting time for action from other stakeholders was more common with increasing age of the physicians, with the oldest age group (55 years or older) reporting it the most. However, older physicians were less likely to conduct such certification due to patient-related factors.

For all types of reasons for issuing sickness certificates for longer periods than necessary, it was more often reported by those physicians that handled certification consultations more than 6 times a week. Certifying sickness certificates for longer periods than necessary due to physician-related factors were less often reported among participants who worked at a workplace with a well-established policy for sickness certifications.

Lack of time and frequency of experiencing delicate situations with patients were related to increased likelihood of reporting issuing unnecessary long sickness absences for all reasons. Frequency of collaboration, referrals, and support in the sickness certification process was related to increased likelihood of issuing sickness certificates for longer periods than necessary due to limitations in the health care system, wait for actions from other stakeholders, and patient factors. Perceived need for more education was related to higher reporting of issuing of sickness certificates for longer periods than necessary due to physician-related factors. Perceived problems with administration were related to issuing of sickness certificates for longer periods than necessary due to limitations in the health care system. Perceived problems with assessments were related to all types of unnecessarily extended sickness certificates except for physician-related factors.

The differences between clinics in frequency of sickness certificates issued for longer periods than necessary could to a large extent be explained by the factors described above. This is demonstrated by the lowered odds ratios, and reduced number of significant differences between clinics in the multivariate analyses. However, some differences still remained between clinics.

## Discussion

The optimal duration of sickness absence is challenging to assess as it varies substantially for various diagnoses and is influenced by situational, individual, and work-related factors. However, the current study indicates that physicians themselves issue sickness certificates for longer periods than they actually deem necessary frequently at some types of clinics. This is of great concern considering the social, economic, and individual implications of extended sickness absences. To the best of our knowledge, this is the first study specifically looking at issuing of sickness absences for longer periods that actually necessary among different medical specialties.

The most common reason for issuing sickness absences for longer periods than justified was factors related to the health care sector such as waiting times for further investigations and treatments. However, the frequency varied substantially between different type of clinical settings, with very high frequency among occupational health, orthopedic, and primary health care physicians; and rather low frequency among infection, oncology, child and youth, ear/nose/throat, dermatology, and eye physicians.

The second most common reason for issuing sickness absences for longer periods than justified was factors related to waiting times for action from other stakeholders such as the Sickness Insurance Agency, employers, or unemployment office. For many of the types of clinics this was reported infrequently, but among occupational health and primary health care physician it was quite frequently reported.

Extended sickness certifications due to non-medical patient factors (i.e. patient do not follow recommendations regarding treatment and rehabilitation) or physician-related factors (i.e. trying to avoid a conflict with the patient, or lack of time to discuss alternatives to sick leave during the medical consultations) were more seldom reported. These reasons for issuing sickness absences for longer periods than necessary were particularly low among physicians working at clinics where they primarily meet patients with medically more definable ‘diseases’ (e.g. oncology, ear/nose/throat, surgery, infection, and dermatology), as compared to physicians working with more symptom-based illnesses (e.g. psychiatry, primary health care, occupational health, and pain-related fields). However, we have no information regarding type of patients or disorders.

In the current study we did not find any gender difference in reporting sickness certification for longer periods than necessary. This is not in line with two studies indicate that female physicians certify sickness absences more than male physicians [15,16], however, several other previous studies have not found such gender differences [17-19]. In the current study we found increasing age to be related to higher degree of certification of sickness absences for longer time than necessary due to waiting time for actions from other stakeholders, and lower degree of such certifications due to patient factors. These associations were found significant in both the univariate and multivariate analyses, and thus, seem to be of importance no matter what type of clinic the physician works at. It is possible that older more experienced physicians have different types of patients requiring more collaboratively organized rehabilitation efforts, and thus, end up being unnecessary on sick leave due to waiting times. Likewise, a more experienced physician might be more skilled in communicating the importance of following self-care instructions, and thus, end up being less frequently on unnecessary sick leave due to patient factors. A previous review of studies on physician factors related to sickness certification found mixed results regarding age and likelihood of certifying sickness absences [20].

Having a well-established workplace policy regarding sickness certification was related to lower number of sickness absences issued for longer time than necessary due to physician factors. This is encouraging and supports the importance of having workplace policies, as well as, efforts of having these policies established at the workplace. However, perceiving support from the immediate manager was not associated with unnecessarily extended sickness certifications.

Frequency of problems, lack of time, delicate interactions with patients, and need for more competence were all related to certifying sickness absences for longer periods than necessary. Differences between clinics in frequency of sickness certificates issued for longer periods than necessary might to a large extent be explained by these factors. This is indicated by the lowered odds ratios, and reduced number of significant differences between clinics in the multivariate analyses. However, some differences still remained between clinics in the multivariable analyses, most notable the higher frequency of extended sickness certifications among occupational health and primary health care physicians; as well as the higher frequency of extended sickness certifications due to patient factors among physicians at psychiatric clinics.

Among the problems reported by the physicians, most notable were that problems with communication with the patient was related to sickness certificates issued for longer periods than necessary due to physician factors; trying to avoid conflicts with patients and not having time to explain alternatives to sick-leave with the patient was associated with issuing sickness absences for longer periods.

Being sickness absent can have a number of serious consequences. The cost of governmental compensation for people on sick leave is extensive, and being on sick leave reduces the available income of the sick-listed individual and can result in reduction of his/hers wellbeing. Further, resent research has given us increased understanding of possible negative effects of being on sick leave for longer periods of time for future sickness absence and mental and physical health [21-26]. This study points out potential targets for interventions trying to decrease unnecessary issued sickness absences. Actions to reduce waiting times within the health care system, as well as, waiting times among other stakeholders involved in the sickness absence process could reduce this type of sickness absence. Further, providing physicians with more time for their work with sickness certifications, communication training, and work-place polices regarding sickness certification practices, are other ways that could reduce unnecessarily issued sickness certificates.

### Limitations

The major strengths of this study are the large number of participants and the fact that all physicians working in Sweden were invited to participate. Other strengths are the many detailed questions about sickness certification tasks. The development of the questionnaire was based on several different previous interview studies (individual and focus groups), questionnaires, discussions, and literature reviews about physicians’ sickness certification practices. However, the study suffers from the limitations associated with self-report, including common method variance and having interpreted the questions in different ways. The later certainly is relevant for the wide question regarding ‘sickness certifying for longer period than actually necessary’. Future studies of the validity of measures regarding physicians’ experiences and problems with sickness certification are warranted. An additional limitation is that the questions regarding frequency of issuing sickness certificates for longer time than actually necessary, only included reasons that were specified in advance. There were no open-ended question were the respondent could indicate alternative reasons not specified in the questionnaire. Thus, it is possible that other reasons than those included in the present study are important for certifying sickness absence for longer time than medically justified. However, in previous studies of our own or published by others, nor in the open-ended comments (about 5000) given, there were no indication of such alternative reasons. As with any cross-sectional study, the design of the study limits our ability to make any conclusions regarding causality. Alternative models and explanation to our findings cannot be ruled out.

## Conclusions

This study showed that physicians experience issuing sickness certificates for longer periods than medically justified quite frequently at some types of clinics. Main reasons for issuing unnecessarily long sick-notes were waiting times for medical investigations, further treatments, and action from other stakeholders involved in the insurance process. Differences between clinics in extended sickness certificates could to a large extent by explained by frequency of problems, lack of time, delicate interactions with patients, and need for more competence.

## Competing interests

The authors declare that they have no competing interests.

## Authors’ contributions

RB, corresponding author, have had the main responsibility for design, analyses, and preparation of the manuscript, LK assisted in statistical issues related to the study. BA, GHN, TL, and KA were involved in the process of planning and developing of the questionnaire, as well as drafting of the manuscript. All authors have read and approved the final manuscript.

## Pre-publication history

The pre-publication history for this paper can be accessed here:

http://www.biomedcentral.com/1471-2458/13/478/prepub
